# An *in vitro* affinity-based method for studying herb–drug interactions for direct identification of cytochrome P450 1A2, 3A4, and 2C9 specific ligands from herbal extracts using ultrafiltration-high performance liquid chromatography†‡

**DOI:** 10.1039/c7ra12161j

**Published:** 2018-02-28

**Authors:** Zhiqiang Wang, Seung Hwan Hwang, Guanglei Zuo, Set Byeol Kim, Soon Sung Lim

**Affiliations:** College of Public Health, Hebei University Baoding 071002 China; Department of Food Science and Nutrition, Hallym University 1 Hallymdeahak-gil Chuncheon 24252 Republic of Korea limss@hallym.ac.kr +82-33-251-0663 +82-33-248-2144; Institute of Korea Nutrition, Hallym University 1 Hallymdeahak-gil Chuncheon 24252 Republic of Korea; Institute of Natural Medicine, Hallym University 1 Hallymdeahak-gil Chuncheon 24252 Republic of Korea

## Abstract

A novel *in vitro* strategy for affinity-based ultrafiltration-high performance liquid chromatography (HPLC) was developed for the direct identification of cytochrome P450 (CYP) 1A2, 3A4, and 2C9 specific ligands from Danshen extracts, in which human liver microsome (HLM) was used as the source of CYP enzymes. The Danshen extract was incubated without HLM, with HLM, or with HLM where the active site of the target enzyme was blocked with a specific competitive probe before ultrafiltration to isolate ligand–enzyme complexes from unbound compounds. Subsequently, HPLC analysis was performed on the released ligands from the complexes. α-Naphthoflavone, ketoconazole, and sulfaphenazole were used as specific competitive probes for CYP1A2, 3A4, and 2C9, respectively. The signal-to-noise ratio (S/N) and specific-signal-to-noise ratio (S-S/N) of each compound were calculated using the obtained peak areas. Finally, two criteria were applied to select putative ligands for each specific target: (1) S/N > 1; (2) S-S/N > 0. Finally, dihydrotanshinone was identified as a specific ligand for CYP1A2 and tanshinone I, cryptotanshinone, and tanshinone IIA were identified as specific ligands for CYP1A2, 2C9, and 3A4. It was demonstrated that the newly developed method can be used to rapidly and directly detect specific ligands from natural product extracts in multi-target systems.

## Abstract 2

The specific ligands in natural product extracts could be identified from a multi-target system by ultrafiltration-high performance liquid chromatography using competitive probes.

## Graphical abstract

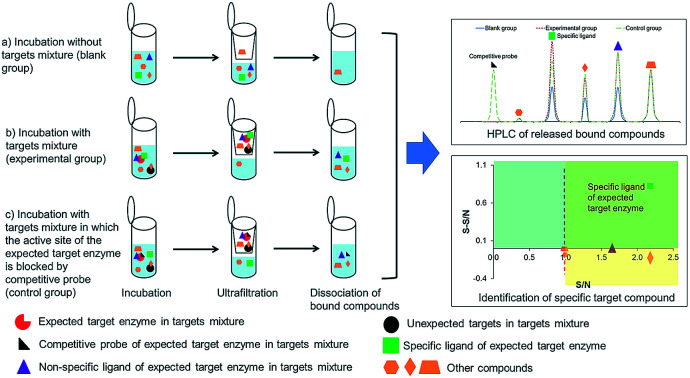

## Introduction

Since the use of herbal medicines has become increasingly popular for the treatment of various diseases in the past two decades, the safety of the co-administration of such products with conventional drugs should be evaluated, because herb–drug interactions may occur, which could lead to adverse outcomes through phytochemical-mediated inhibition or induction of hepatic drug-metabolizing enzymes.^1,2^ Although the pharmacokinetics and pharmacodynamics of conventional drugs are well-established, co-administered herbal products have not been sufficiently investigated due to their complex components and variability. To predict the metabolism-based interactions between drugs and herbal products, the components of herbal products as well as their effects on the activities of hepatic drug-metabolizing enzymes should be identified.^3,4^ Unfortunately, the chemical complexity of herbal products makes understanding the interactions of each component with the related drug-metabolizing enzyme impossible before isolation, regardless of the method used (*in vitro* or *in vivo*).

Affinity-based ultrafiltration-high performance liquid chromatography (HPLC) has emerged as a rapid and convenient approach for protein–ligand interaction studies, and has been widely applied for the screening and identification of ligands of enzymes, proteins, or receptors from natural product matrices, in which small molecules from natural product extracts bound to selected targets (enzymes, proteins, or receptors) can be separated from unbound small molecules by ultrafiltration and subsequently identified.^5^ Thus, examination of the HPLC profile of certain peaks can be used to identify “hits”. Affinity-based ultrafiltration-HPLC has significant advantages including rapid and convenient operation, low cost, high hit ratio, and lack of immobilization of screening targets on a carrier as in other active molecular screening methods such as bioassay-guided fractionation, biochromatography, magnetic separation, microdialysis, hollow fiber adsorption *etc.*^6–8^ Even though the disadvantages of affinity-based ultrafiltration-HPLC such as false positive results and false negative results can be avoided,^9^ high concentrations of pure screening targets (enzymes, proteins, or receptors) are required.^10^ To our knowledge, only a small proportion of commercially available pure targets (enzymes, proteins, or receptors) are cheap and stable, and most are expensive; many targets cannot be prepared easily and are not commercially available. The use of tissue products (*i.e.*, microsomes, extracts *etc.*) or cell lysates is more feasible and cost effective. Nevertheless, previous studies have only described multi-ligand screening in single-target systems on affinity-based ultrafiltration-HPLC platforms, and using this method for screening in a multi-target system is still a challenge.

Herein, we propose a novel strategy for affinity-based ultrafiltration-HPLC for the detection of specific ligands of multiple targets from natural product extracts using label-free competitive probes. As a proof-of-concept, the specific ligands of cytochrome P450 (CYP) 1A2, 2C9, and 3A4 were directly identified from Danshen (*Salvia miltiorrhiza*) ethanol extracts (DEE), in which human liver microsome (HLM) was used as the source of CYP enzymes instead of pure recombinant human enzymes. The CYP superfamily is an important drug-metabolizing enzyme system and is involved in the biotransformation of a number of exogenous (drugs, toxic chemicals, organic solvents, carcinogens, and environmental pollutants) and endogenous compounds (steroids hormones, fatty acids, bile acids, and prostaglandins). It is almost universally accepted that over 90% of drug metabolism in humans is mediated by CYP enzymes.^11^ Moreover, among the CYPs identified to date, enzymes from CYP1, CYP2, and CYP3 families catalyse the biotransformations of the majority of drugs used in clinics today. Danshen is the dried root and rhizome of *Salvia miltiorrhiza* and is a widely used medicinal plant for the treatment of cardiovascular disease in China and serves as a complementary medicine in the West.^12^ Among the major active constituents of Danshen that have been isolated and characterized, tanshinones have been reported to exhibit anti-platelet, cardio-protective, anti-inflammatory, hepato-protective, and anti-HIV effects in preclinical studies.^13^ Moreover, a number of herb–drug interactions leading to adverse outcomes were reported to involve Danshen when it was co-administered with certain therapeutic agents.^14,15^ As the pharmacokinetics of Danshen have been well studied, it was selected to validate the proposed method.

## Results and discussion

**Fig. 1:**
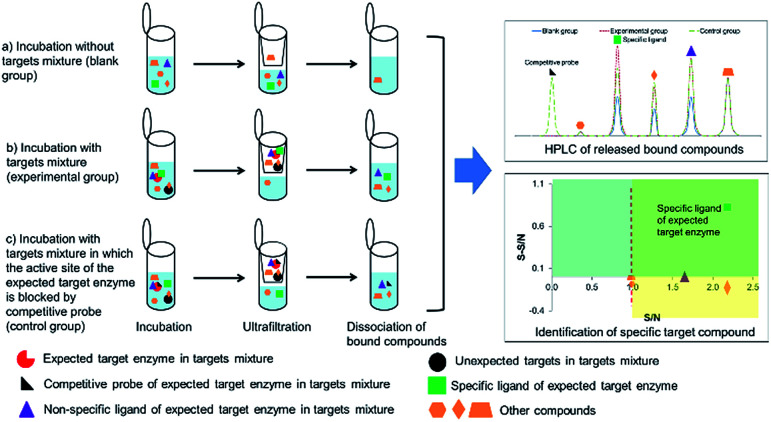
Strategy for identifying specific ligands of expected target enzymes in a multi-target system from herbal extracts using ultrafiltration-high performance liquid chromatography (HPLC) with competitive probes. S/N, signal-to-noise ratio; S-S/N, specific-signal-to-noise ratio.

In order to identify specific ligands for a target enzyme in a multi-target system, a strategy for affinity-based ultrafiltration-HPLC combined with competitive binding was developed. The general procedures are summarized in Fig. 1. The major principles are as follows: a solution of the natural product matrix such as DEE is incubated with the target mixture (*i.e.*, HLM). During the incubation process, ligands in the matrix bind to targets to form complexes, which can be separated from unbound small molecules through the ultrafiltration membrane. The ligands that dissociated from complexes retained on the ultrafiltration membrane were collected and subjected to HPLC analysis to obtain the chromatogram of bound ligands in the mixture. To eliminate non-specific binding induced by interactions between the ligands and ultrafiltration membrane, a blank group was carried out by incubating without the targets mixture before ultrafiltration. To determine the specific ligands, a control group was carried out with incubation in the presence of targets mixture where the active site of the expected target enzyme was blocked with a specific competitive probe before ultrafiltration. According to eqn (1) and (2), the signal-to-noise ratio (S/N) and specific signal-to-noise ratio (S-S/N) of each compound in the natural product extract was calculated. S/N can be used to rank the binding affinities of compounds from natural product extracts towards various targets. S-S/N indicates the competitive binding ability of an expected target enzyme in mixture between the competitive binding probe and ligands. This information was used to develop S/N and S-S/N plots. Finally, two criterial were applied to select putative specific ligands of expected target enzymes in the target mixture: (1) S/N > 1 represents compounds with binding affinities towards the targets; (2) S-S/N > 0, represents competitive binding between the ligand and competitive probe.

**Fig. 2:**
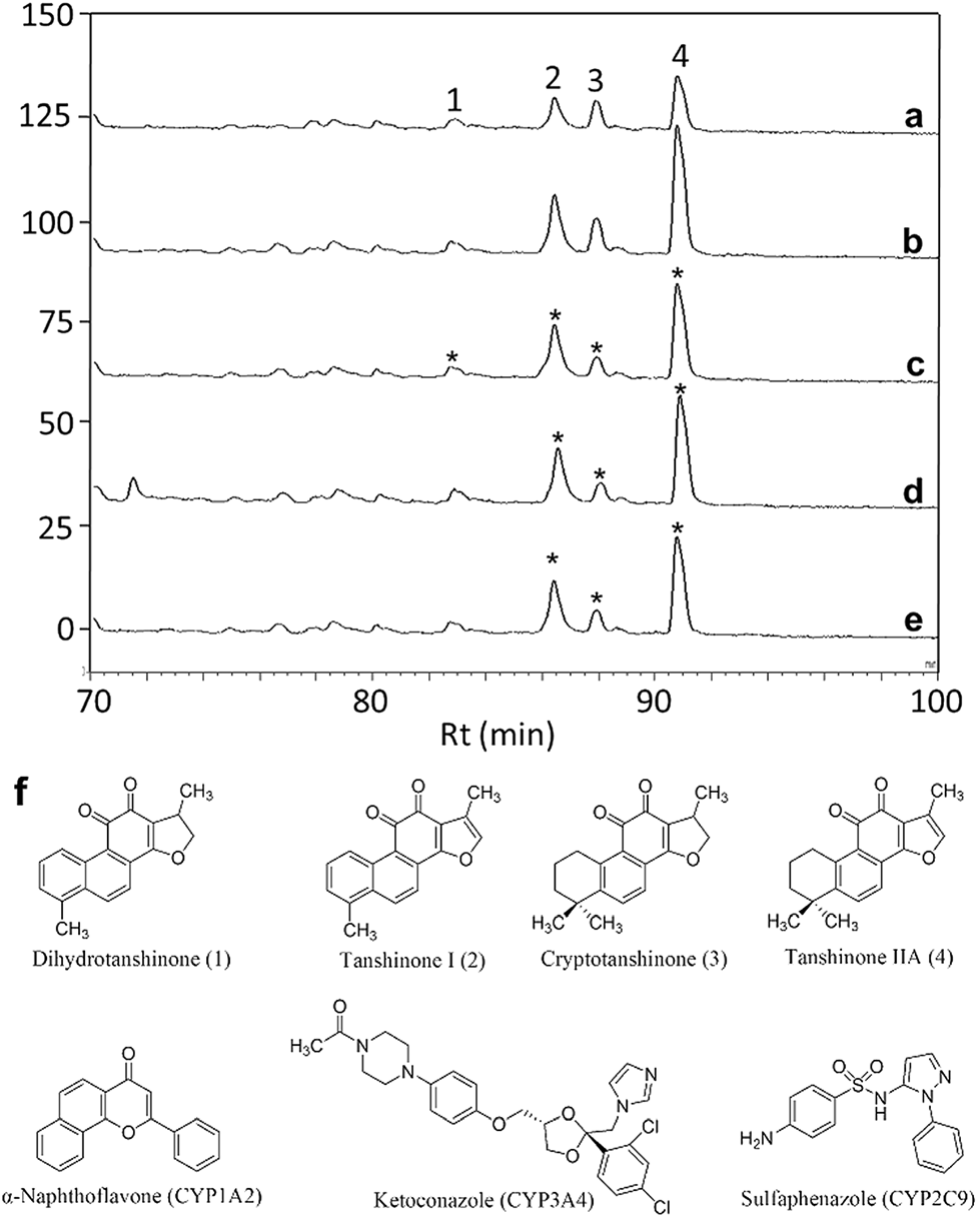
Identification of specific ligands of CYP1A2, 3A4, and 2C9 in human liver microsome (HLM) from Danshen ethanol extract (DEE) using ultrafiltration-high-performance liquid chromatography (HPLC) with competitive probes. (a) Danshen ethanol extract incubated without HLM (blank group); (b) Danshen ethanol extract incubated with HLM (experimental group); (c) Danshen ethanol extract incubated with HLM in which the active site of CYP1A2 was blocked with a competitive probe (control group); (d) Danshen ethanol extract incubated with HLM in which the active site of CYP3A4 was blocked with a competitive probe (control group); (e) Danshen ethanol extract incubated with HLM in which the active site of CYP2C9 was blocked with a competitive probe (control group); (f) structures of identified ligands from Danshen ethanol extract (DEE) and competitive probes. α-Naphthoflavone, ketoconazole, and sulfaphenazole were used as competitive probes for CYP1A2, 3A4, and 2C9, respectively. Asterisk indicates the ligands identified using the present method. Compound 1 is dihydrotanshinone; compound 2 is tanshinone I; compound 3 is cryptotanshinone; compound 4 is tanshinone IIA.

**Fig. 3:**
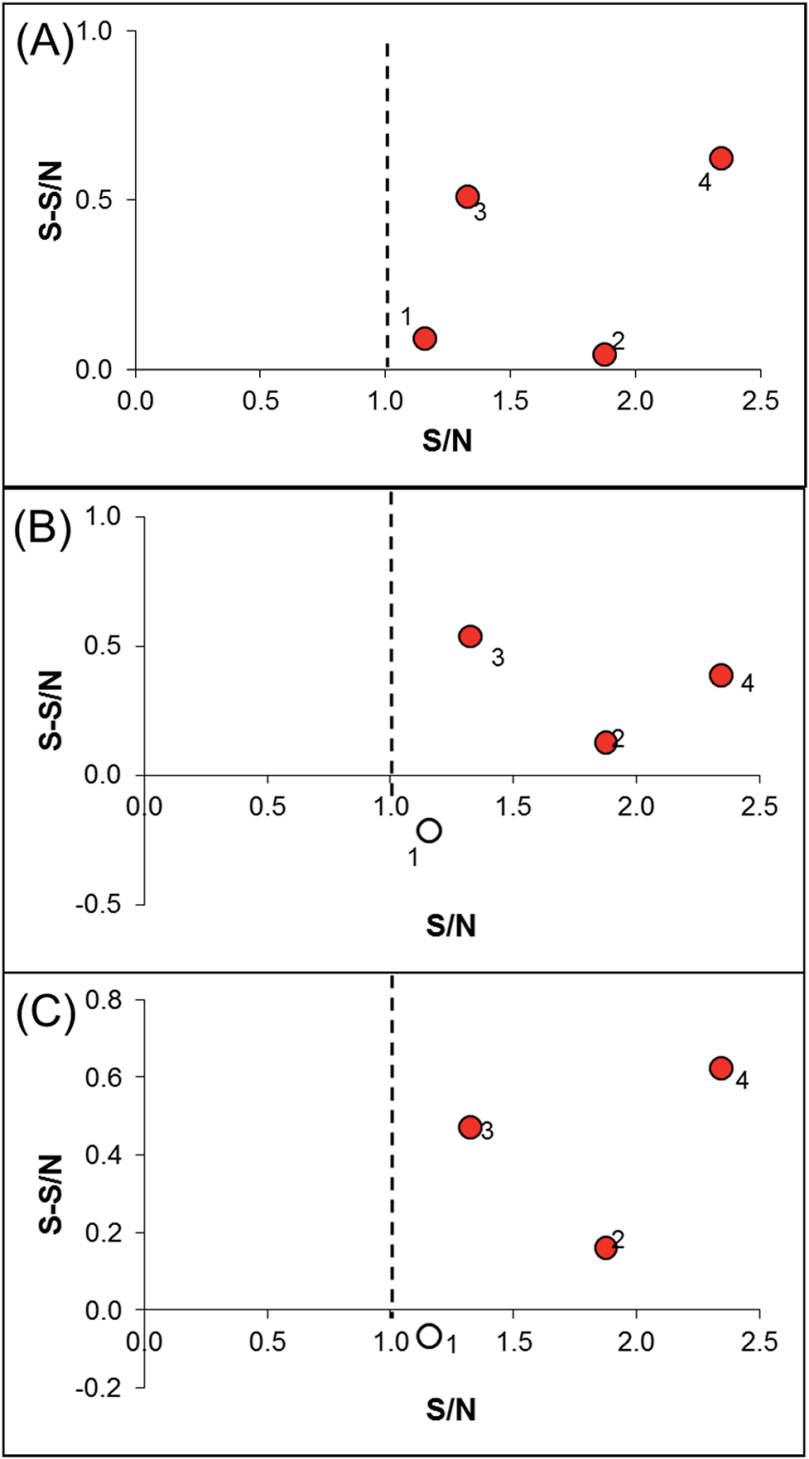
The S/N and S-S/N plots of features detected in ultrafiltration-HPLC with competitive probes. (A) CYP1A2; (B) CYP3A4; (C) CYP2C9. S/N, signal-to-noise ratio; S-S/N, specific-signal-to-noise ratio. Compound 1 is dihydrotanshinone; compound 2 is tanshinone I; compound 3 is cryptotanshinone; compound 4 is tanshinone IIA. Red dots are identified specific ligands.

As a proof-of-concept, a case study was carried out as described previously to directly identify the specific ligands of CYP1A2, 3A4, and 2C9 from DEE, in which HLM was used as the source of CYP enzymes instead of pure recombinant human enzymes. As mentioned previously, Danshen is a popular herbal medicine and is widely used for promoting blood circulation to remove blood stasis, clearing away heat, relieving vexation, nourishing blood, and tranquilizing the mind and cooling blood to relieve carbuncles. Among the major active isolated and characterized constituents of Danshen, tanshinones, a series of abietane diterpenes, have been reported to exhibit anti-platelet, cardio-protective, anti-inflammatory, hepatoprotective, and anti-HIV effects in preclinical studies. The four major tanshinones in Danshen are dihydrotanshinone, tanshinone I, cryptotanshinone, and tanshinone IIA. Numerous studies have demonstrated the potential applications of these tanshinones in a broad spectrum of maladies such as atherosclerosis, cardiac arrhythmias, hypertension, obesity, metabolic syndromes, and cancer. Thus, the potential herb–drug interactions between tanshinones, including dihydrotanshinone, tanshinone I, cryptotanshinone, and tanshinone IIA, with therapeutic agents have been evaluated.^16^ In the present study, α-naphthoflavone, ketoconazole, and sulfaphenazole were selected as competitive probes for CYP1A2, 3A4, and 2C9, respectively (Fig. 2f). According to Fig. 2a–e, the S/Ns and S-S/Ns of compounds from DEE were measured (Table 1), and the S/N and S-S/N plots are shown in Fig. 3. Although a number of compounds from DEE showed binding affinities towards HLM (Fig. 2a and b), only compound 1 (S/N = 1.16; S-S/N_1A2_ = 0.09) was identified as a specific ligand of CYP1A2; compound 2 (S/N = 1.88; S-S/N_1A2_ = 0.04; S-S/N_2C9_ = 0.16; S-S/N_3A4_ = 0.13), compound 3 (S/N = 1.33; S-S/N_1A2_ = 0.51; S-S/N_2C9_ = 0.47; S-S/N_3A4_ = 0.54), and compound 4 (S/N = 2.34; S-S/N_1A2_ = 0.62; S-S/N_2C9_ = 0.62; S-S/N_3A4_ = 0.39) were identified as specific ligands of CYP1A2, 2C9, 3A4. The four compounds were identified as dihydrotanshinone (1), tanshinone I (2), cryptotanshinone (3), and tanshinone IIA (4) (Fig. 2f).

**Table 1:** S/N, S-S/N, IC50, Ki, and inhibition mode of tanshinones on human CYP1A2, 3A4, and 2C9

CYP isoform	Danshen components^a^	S/N^b^	S-S/N^c^	IC_50_^d^ (μM)	*K*_i_ d (μM)	Mode of inhibition^d^
1A2	Dihydrotanshinone (1)*	1.16	0.09	0.50	0.53	Competitive
	Tanshinone I (2)*	1.88	0.04	1.70	2.16	Competitive
	Cryptotanshinone (3)*	1.33	0.51	3.06	1.88	Competitive
	Tanshinone IIA (4)*	2.34	0.62	2.01	1.45	Competitive
3A4	Dihydrotanshinone (1)	1.16	−0.21	3.22	2.11	Non-competitive
	Tanshinone I (2)*	1.88	0.13	>100	86.9	Competitive
	Cryptotanshinone (3)*	1.33	0.54	>100	120.4	Competitive
	Tanshinone IIA (4)*	2.34	0.39	>100	218.7	Competitive
2C9	Dihydrotanshinone (1)	1.16	−0.07	7.48	1.92	Competitive
	Tanshinone I (2)*	1.88	0.16	>100	51.2	Competitive
	Cryptotanshinone (3)*	1.33	0.47	23.86	22.9	Competitive
	Tanshinone IIA (4)*	2.34	0.62	>100	61.6	Competitive

a Asterisk indicated the specific ligands identified by ultrafiltration-HPLC with probes.

b S/N is the signal-noise ratio.

c S-S/N is the specific signal-noise ratio.

d IC_50_, *K*_i_, and mode of inhibition were cited from Wang *et al.*^16^; *K*_i_ is the inhibitory constant.

To validate our results, we summarized the data obtained in this and previous studies using traditional inhibition methods (Table 1). The results revealed that these tanshinones are ligands (inhibitors) of CYP1A2, 3A4 and 2C9. However, these results do not exactly match those obtained with our newly developed approach of ultrafiltration-HPLC with competitive probes, where dihydrotanshinone was not identified as a specific ligand of CYP3A4 and CYP2C9. Originally, competitive binding experiments were used to verify and screening results,^17^ but Chen *et al.* first used competitive binding experiments in ultrafiltration-HPLC to eliminate false positives.^18^ In fact, not only false positive, but also ligands with different binding sites from competitive binding compounds will be eliminated by such competitive binding experiments. Thus, Song *et al.* and Wang *et al.* proposed that competitive binding experiments could be used to identify selective and specific enzyme inhibitors from natural products.^9,19^ Recently, Wang *et al.* concluded that competitive binding experiments could reduce the false positive results of affinity-based ultrafiltration-HPLC.^20^ Alternatively, in multi-target systems, all ligands binding to unexpected targets can be considered false positive results, and the specific ligand of the expected target enzyme might be distinguished by competitive binding experiments by adding a specific competitive probe compound. We proposed the developed method under this assumption. However, one limitation of competitive binding experiments is that not all false positives can be eliminated and some ligand candidates may be excluded, similar to the results obtained herein. Wang *et al.* indicated that this limitation is due to the properties of the competitive binding compounds, including binding site and affinity differences between ligands and competitive probes.^20^ Put simply, the developed strategy will only work when binding site of the ligand, which has a similar or lower binding affinity than the competitive binding probe. Thus, selecting an appropriate competitive probe is critical. Dihydrotanshinone was not identified as a specific ligand for CYP3A4 and CYP2C9 perhaps because dihydrotanshinone has a different binding site than the probes or has a relatively high binding affinity towards the target enzyme.

**Fig. 4:**
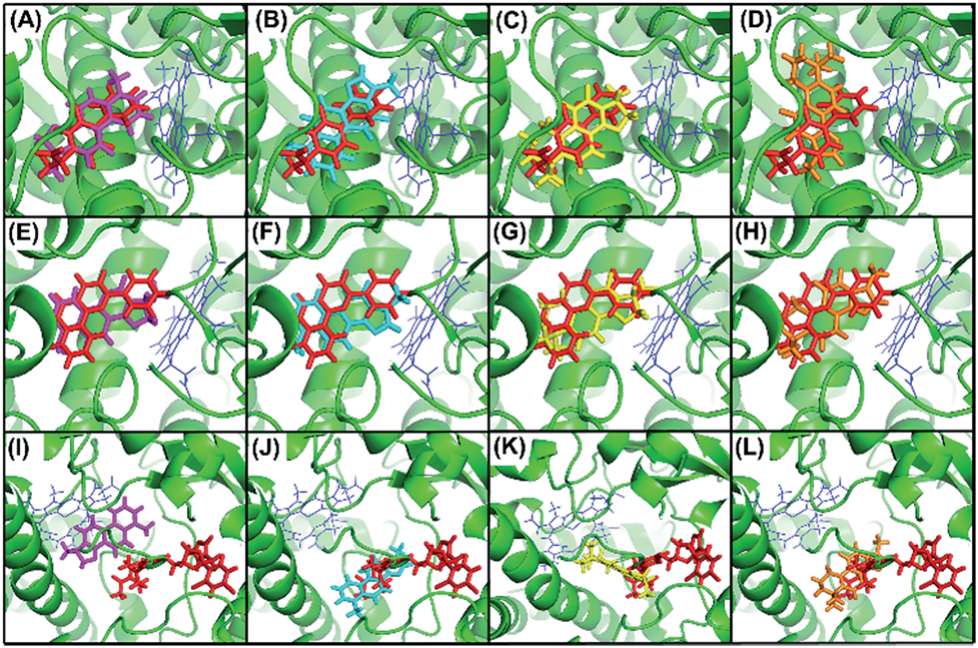
Docking model of dihydrotanshhinone (magenta), tanshinone I (cyan), cryptotanshinone (yellow), and tanshinone IIA (orange) to CYP1A2 (A–D), 2C9 (E–H), and 3A4 (I–L) with competitive probes (red). α-Naphthoflavone, sulfaphenazole, and ketoconazole were used as competitive probes for CYP1A2 (A–D), 2C9 (E–H), and 3A4 (I–L), respectively.

To confirm our hypothesis, the results of the *in vitro* kinetic analysis and *in silico* computer simulation of docking model are presented in Table 1 and Fig. 4. The kinetic results (Table 1) revealed that dihydrotanshinone is a competitive inhibitor of CYP1A2 (*K*_i_ = 0.53 μM) and CYP2C9 (*K*_i_ = 1.92 μM) and is a non-competitive inhibitor of CYP3A4 (*K*_i_ = 2.11 μM); tanshinone I is a competitive inhibitor of CYP1A2 (*K*_i_ = 2.16 μM), CYP2C9 (*K*_i_ = 51.20 μM), and CYP3A4 (*K*_i_ = 86.90 μM); cryptotanshinone is a competitive inhibitor of CYP1A2 (*K*_i_ = 1.88 μM), CYP2C9 (*K*_i_ = 22.90 μM), and CYP3A4 (*K*_i_ = 120.40 μM); tanshinone IIA is a competitive inhibitor of CYP1A2 (*K*_i_ = 1.45 μM), CYP2C9 (*K*_i_ = 61.60 μM), and CYP3A4 (*K*_i_ = 218.70 μM). These results suggested that (1) dihydrotanshinone might be a specific, high affinity ligand of CYP2C9 because it exhibited competitive inhibition and a much lower *K*_i_ value than the other three compounds; (2) dihydrotanshinone might be a non-specific, high affinity ligand of CYP3A4 because it showed non-competitive inhibition and a much lower *K*_i_ value than the other three compounds. The computer simulation results (Fig. 4) revealed that all tanshinones had the same binding site as the probes except dihydrotanshinone and ketoconazole for CYP3A4, indicating that dihydrotanshinone is not a specific ligand of CYP3A4. These results demonstrated that the developed method can be used to rapidly and directly identify specific ligands from natural product extracts in multi-target systems, and the selection of appropriate competitive probes is critical in this method.

## Materials and methods

### Reagents

HLM (total protein concentration is 20 mg mL^−1^) and 0.5 M potassium phosphate buffer (pH 7.4) were purchased from Corning (New York, NY, USA). α-Naphthoflavone, ketoconazole, sulfaphenazole, dimethyl sulfoxide (DMSO), and trifluoroacetic acid (TFA) were obtained from Sigma-Aldrich (St. Louis, MO, USA). All organic solvents were of HPLC-grade and were obtained from J. T. Baker (Phillipsburg, NJ, USA). Ultrapure water with a resistivity over 18.2 MΩ cm, generated by a Milli-Q laboratory water purification system (Millipore, Billerica, MA, USA), was used for all solutions, dilutions, and HPLC analyses. All other reagents, unless otherwise specified, were purchased from Sigma-Aldrich as were used as received without further purification.

### Preparation of plant extracts and stock solutions

Danshen was purchased from a local market in Chuncheon, Korea. A voucher sample (RIC-2012-5) was deposited at the Center for Efficacy Assessment and Development of Functional Foods and Drugs, Hallym University in Chuncheon, Korea. Danshen was extracted 3 times with ethanol for 3 h. The solvent was evaporated under reduced pressure below 45 °C to give the ethanol extract.

The DEE was dissolved in DMSO to prepare the extract sample solution (1 mg mL^−1^) for further assays. The HLM was dissolved in potassium phosphate buffer (0.5 M, pH 7.4) to prepare HLM potassium phosphate buffer stock solution (5 mg mL^−1^) for further assays. The probes including α-naphthoflavone, ketoconazole, and sulfaphenazole were dissolved in 20% methanol (v/v) to prepare the probe stock solutions (5 Mm) for the competitive binding experiments.

### Ultrafiltration procedures

30 μL of DEE sample solutions, 6 μL of HLM potassium phosphate buffer stock solution, 30 μL of 20% methanol water solution (v/v), and 234 μL of 0.5 M potassium phosphate buffer (pH 7.4) were mixed and incubated at 37 °C for 60 min. The incubated mixture was filtered through a Microcon YM-10 centrifugal filter unit with a 10 000 MW cut-off membrane (Millipore) by centrifugation at 6350 × *g* for 30 min at 4 °C, followed by two washes with potassium phosphate buffer to remove unbound compounds. Meanwhile, the protein complexes retained on the membrane of the ultrafiltration unit were transferred to a new centrifugal tube. The ligands were dissociated from the protein complexes with 50% methanol (v/v) and were separated from the denatured proteins by centrifugation, and were subsequently washed with 50% methanol (v/v). The released bound ligands were combined and dried under nitrogen gas for 3 h. Next, the dried bound compounds were reconstituted in 50% methanol (v/v, 100 μL) and the reconstituted bound fractions were subjected to HPLC analysis using the methods described in Section “HPLC conditions”. The blank experiment was carried out similarly, but without the addition of probe pre-incubated HLM, which was prepared following the methods described in Section “Competitive binding experiments”. To rank the binding affinities of the ligands to HLM, the S/N was calculated using eqn (1):

1 S/N = *A*_a_/*A*_b_

where *A*_a_ and *A*_b_ are the HPLC peak areas of released bound ligands from samples incubated with and without HLM, respectively.

### Competitive binding experiments

The competitive binding experiments were carried out using the probe pre-incubated HLM. α-Naphthoflavone was used as the competitive probe for CYP1A2, sulfaphenazole was used as the competitive probe for CYP2C9, and ketoconazole was used as the competitive probe for CYP3A4. For the pre-incubation of HLM with each specific probe, 60 μL of a 5 mg mL^−1^ HLM potassium phosphate buffer (0.5 M, pH 7.4) stock solution was incubated with 30 μL of a 5 mM probe stock solution at 37 °C for 1 h. Then, the probe pre-incubated HLM and extract sample solution (final concentration of 0.1 mg mL^−1^) were mixed in a total volume of 300 μL and incubated at 37 °C for 1 h. The incubated mixtures were subjected to ultrafiltration and HPLC analysis following the methods described in Sections “Ultrafiltration procedures” and ‘HPLC conditions”. The S-S/N was defined as the competitive binding ability of specific CYP enzymes in HLM between its specific probe and ligands. S-S/N can be calculated using eqn (2):

2 S-S/N = (*A*_a_ − *A*_c_)/*A*_b_

where *A*_a_, *A*_b_, and *A*_c_ are the HPLC peak areas of released bound ligands incubated with HLM, without HLM, and with probe pre-incubated HLM.

### HPLC conditions

HPLC analysis was performed on a Dionex system (Dionex, Sunnyvale, CA, USA) comprised of a P850 pump, an ASI-100 automated sample injector, STH585 column oven, and UVD170S detector. Separation was achieved on an Eclipse Plus C18 column (150 mm length, 4.6 mm i.d., and 5 μm particle size; Agilent, Santa Clara, CA, USA), coupled with a guard column at 30 °C. Briefly, 25 μL of the prepared ligand solution was injected into the system and eluted with acidified water (0.1% TFA, A) and methanol (B) at a flow rate of 0.7 mL min^−1^. The optimized gradient chromatography conditions were 5–100% B from 0–105 min. The eluent was monitored at 254 nm.

### Computer simulations of binding by molecular docking

We used the Surflex-dock software for molecular docking analysis. The 3D crystal structures of human CYP1A2 (in complex with α-naphthoflavone), 2C9 (in complex with flurbiprofen), and 3A4 (in complex with ketoconazole), solved at resolutions of 1.95 Å, 2.0 Å, and 2.8 Å, respectively, were obtained from the RCSB Protein Data Bank (accession id: 2HI4 for CYP1A2; 1R9O for CYP2C9; 2V0M for CYP3A4). The protein preparation wizard in the Sybyl software was used to remove non-protein atoms and water molecules from PDB files, to correct structural defects in the raw structure such as missing atoms and residues, and for energy minimization. The probe and ligand structures were constructed using Sybyl. Prior to docking, the probe and ligand molecules were optimized using the Tripos force field and MMFF94 partial charges. The “thresh” and “bloat” were set to 0.5 and 1 for generation of a protomol, and the number of additional starting conformations and resulting docking poses were set to 5 to 20, respectively. To validate the docking process, the redocking of enzymes with the inhibitors which are originally in the complexes downloaded from RCSB Protein Data Bank were performed, and the root-mean-square deviation (RMSD) for each enzyme was provided in the ESI.‡ The obtained total scores of the molecular docking is shown in Table S2.‡ The 3D molecular structure was visualized with PyMOL.

## Conclusions

In conclusion, a novel *in vitro* strategy for affinity-based ultrafiltration-HPLC with competitive binding probes for the direct identification of specific ligands in multi-target systems from natural products was proposed. Using CYP1A2, 3A4, and 2C9, specific ligands from Danshen extracts, where HLM was used as the source of CYP450 and α-naphthoflavone, ketoconazole, and sulfaphenazole were used as specific competitive probes, we concluded that the newly developed method can be used for the identification of specific ligands from natural product extracts in multi-target systems.

## Conflicts of interest

There are no conflicts to declare.

## Supplementary Material

RA-008-C7RA12161J-s001
