# Supplementary material for: An in vitro affinity-based method for studying herb–drug interactions for direct identification of cytochrome P450 1A2, 3A4, and 2C9 specific ligands from herbal extracts using ultrafiltration-high performance liquid chromatography
Source: RSC Adv. 2018 Feb 28;8(16):8944–9. doi: 10.1039/c7ra12161j (PMC9078662; doi:10.1039/c7ra12161j)
Supplement: RA-008-C7RA12161J-s001 [file RA-008-C7RA12161J-s001.pdf]

Electronic Supplementary Material (ESI) for RSC Advances.
This journal is © The Royal Society of Chemistry 2018

Supplementary materials

Table S1. The root-mean-square deviation (RMSD) for CYP1A2, 2C9, and 3A4.

CYP isoform	RMSD
1A2	0.413931
2C9	0.020090
3A4	1.454134

Table S2 The total scores of CYP isoforms-ligands molecular docking

CYP isoform	Probe[1]	Dihydrotanshinone	Tanshinone I	Cryptotanshinone	Tanshinone IIA
1A2	8.1835	8.7425	8.6532	5.9218	6.4312
2C9	5.0936	3.5947	5.0060	2.6552	4.0515
3A4	16.4210	4.4355	3.8878	6.1238	4.9618

[1]α-Naphthoflavone, sulfaphenazole, and ketoconazole are probes for CYP 1A2, 2C9, and 3A4, respectively.
